# Active listing and more consultations in primary care are associated with shorter mean hospitalisation and interacting with psychiatric disorders when adjusting for multimorbidity, age and sex

**DOI:** 10.1080/02813432.2018.1499514

**Published:** 2018-09-21

**Authors:** Karin Ranstad, Patrik Midlöv, Anders Halling

**Affiliations:** a Department of Clinical Sciences in Malmö, Center for Primary Health Care Research, Lund University, Lund, Sweden;; b County of Blekinge, Nättraby Primary Health Care Centre, Nättraby, Sweden

**Keywords:** Hospitalization, primary health care, health services research, multimorbidity, mental disorders

## Abstract

**Objective:** Patient-provider relationships with primary care and need for hospitalisations are related within the complex networks comprising healthcare. Our objective was to analyse mean days hospitalised, using registration status (active or passive listing) with a provider and number of consultations as proxies of patient-provider relationships with primary care, adjusting for morbidity burden, age and sex while analysing the contribution of psychiatric disorders. The Johns Hopkins Adjusted Clinical Groups Case-Mix System was used to classify morbidity burden into Resource Utilization Band (RUB) 0-5.

**Design:** Cross-sectional population study using zero-inflated negative binomial regression.

**Setting and subjects:** All population in the Swedish County of Blekinge (*N* = 151 731) in 2007.

**Main outcome measure:** Mean days hospitalised.

**Results:** Actively listed were in mean hospitalised for 0.86 (95%CI 0.81–0.92) and passively listed for 1.23 (95%CI 1.09–1.37) days. For 0–1 consultation mean days hospitalised was 1.16 (95%CI 1.08–1.23) and for 4–5 consultations 0.68 (95%CI 0.62–0.75) days. At RUB3, actively listed were in mean hospitalised for 3.45 (95%CI 2.84–4.07) days if diagnosed with any psychiatric disorder and 1.64 (95%CI 1.50–1.77) days if not. Passively listed at RUB3 were in mean hospitalised for 5.17 (95%CI 4.36–5.98) days if diagnosed with any psychiatric disorder and 2.41 (95%CI 2.22–2.60) days if not.

**Conclusions:** Active listing and more consultations were associated with a decrease in mean days hospitalised, especially for patients with psychiatric diagnoses.

**Implications:** Promoting good relationships with primary care could be an opportunity to decrease mean days hospitalised, especially for patients with more complex diagnostic patterns.

**Key Points**
- Primary care performance, patient-provider relationships and need for hospitalisation are related within the complex networks comprising healthcare systems.
- Good patient-provider relationships, i.e. more consultations and active listing, with primary care are associated with decreasing mean days hospitalised.
- The impact of patient-provider relationships in primary care on mean days hospitalised increased when psychiatric disorders added to patient complexity.

## Introduction

Need for hospital admissions increases with severity of morbidity and decreases with adequate out-of-hospital care [1,2]. Psychiatric disorders are related to multimorbidity, patient complexity, and are associated with common somatic disorders, e.g. chronic obstructive pulmonary disease and diabetes [2–4]. Managing clusters of conditions where management of one disease will also benefit management of another disease is potentially simpler than dealing with clusters of non-synergistic conditions. When psychiatric disorders add to multimorbidity they are associated with health-related outcomes [5]. Increased morbidity burden, associated with age and sex, increases risk of hospital admissions. Psychiatric disorders are correlated with morbidity burden and patient complexity, and are related to use of healthcare and trust. Trust is related to relationships and availability of healthcare services [6].

The connection between availability of primary care and hospital admission has been studied extensively. A review showed that better access to quality primary health care results in fewer hospital admissions for ambulatory care sensitive chronical conditions [7]. Primary care is consulted for co-occurring disorders, even when the main disease requires advanced specialist care. Both the generalistic approach and personalised care of primary care are facilitated by long-term personal relations [8,9]. Within the complex networks comprising healthcare systems, patient-professional relationships connect distant parts of healthcare, with relationships as the central unit of analysis [10]. Mean days hospitalised could then be analysed as an outcome of relationships in primary care [11].

In Sweden, primary care practices comprise general practitioners (GPs) organised within multidisciplinary teams. County councils regulate local healthcare and organise primary care into several quasi-market models [12,13]. Listing, i.e. enrolment or registration, was introduced to empower patients and to introduce market models. Within the studied population active listing might be considered an act of patients to protect their relationship with primary care and number of consultations as using this relationship while adjusting for morbidity.

To our knowledge, it has not been studied before whether mean days hospitalised is related to patient-professional relationships in primary care, accounting for both morbidity burden and the additional challenge of mental health problems increasing patient complexity. We study mean days hospitalised and odds of any hospital admission for a Swedish population. The hypothesis is that listing status and number of consultations are associated with mean days hospitalised and that more psychiatric disorders increase mean days hospitalised compared to patients without psychiatric disorders with the same need for health care. Our aim is to analyse how relationships in primary care, and more complex patterns of disorders are associated with mean days hospitalised, adjusting for morbidity burden, age and sex.

## Material and methods

### Study population and settings

Year 2007 represents a stable period in Swedish primary care. Settings in the county of Blekinge were stable regarding funding, regulation and workforce. On 31 December 2007 the county had 151,731 inhabitants. Of these, 50.5% were men and the average age was 42.7 years [14]. Health care was provided by 2 hospitals, 5 psychiatric clinics and 25 primary care practices. A total of 65% of the population was actively listed, and the prevalence of diagnosed psychiatric disorder 4.7%. Mean number of consultations in primary care was 0.9, and 0.6% consulted a GP more than seven times that year.

A listing (i.e. enrolment or registration) system, was introduced in primary care in Blekinge in 2004 with active or passive listing as the only options. Listing was mandatory passive at the nearest primary care practice. Patients could change listing to active at will, at the same practice or another within the county, by notifying the practice of choice. Active listing was owned exclusively by patients with no availability or access to gain but given a possibility to state their relationship with a specific GP practice. Family members over 15 years of age made their choices individually. Access and availability to primary care were the same regardless of listing status. Primary care practices were obliged to accept any listed patient and to distribute care according to medical needs. Regulations were the same for all practices, and there were no incentives to treat actively listed patients different from passively listed.

### Design

We performed a cross-sectional population-based study on mean days hospitalised as an outcome of patient-provider relationships with primary care. Listing status and number of consultations were used as proxies of relationships with primary care, and the contribution of more complex diagnose patterns including psychiatric disorders was investigated. We adjusted for morbidity burden, age and sex. Data collected from electronic patient records during year 2007 was used. The Regional Ethical Review Board at Lund University (application no. 2016/71) approved the study.

### Outcomes

Mean days hospitalised for the population were predicted from a count regression model showing odds of any admission to hospital and mean number of days hospitalised if at risk of hospitalisation, in all health care (somatic and psychiatric care) in Blekinge County during year 2007.

### Explanatory factors

Listing status (actively or passively listed) in primary care, active listing considered a proxy of good relationship in primary care due to the settings and use of the listing system.

Number of consultations in primary care was used to quantify how the patient-professional relationship in primary care was used. More than the mean of 0.9 consultations (two or more to be conservative) with a doctor (GP) was considered to be associated with having a relationship when adjusting for morbidity burden. Consultations with a GP was categorised into five groups (0–1, 2–3, 4–5, 6–7, 8- consultations).

Psychiatric disorders, using the International Statistical Classification of Diseases and Related Health Problems diagnoses (ICD 10, F00–F99), were categorised as psychoses (F20–F29), depressive disorders (F30–F39), anxiety disorders (F40–F48), and others (F00–F19 and F49–F99). When needed for statistical validity, diagnosed with any psychiatric disorder was used.

Morbidity burden during 2007 was estimated using all diagnoses from electronic patient records from all health care, using the Johns Hopkins Adjusted Clinical Groups Case-Mix System (ACG). This is one of the summary measures aiming to link diagnoses with need of healthcare. These measures focus on stratification or classification of patients into groups according to diseases and conditions, age and sex. ACG weighted patients’ diagnoses according to five clinical dimensions: duration, severity, diagnostic certainty, aetiology and need for specialist care into almost 100 mutually exclusive ACGs. Then ACGs were categorised into six multimorbidity levels with similar impact on need for healthcare despite different patterns of diagnoses. These multimorbidity levels, called Resource Utilization Bands (RUBs), ranged from 0 (no need for health care) to 5 (very strong need for health care) [15–17].

Age and sex, age grouped into (0–19, 20–39, 40–59, 60–79, 80- years of age).

The statistical analysis was clustered at primary care practice (25 practices).

### Statistical analysis

Statistical analyses were performed with STATA version 14.0 (Stata Corporation, Texas, USA). Associations between variables were studied using pairwise correlations, univariate and multivariate statistics. Number of days hospitalised was found to be significantly skewed, non-normally distributed and with considerable over-representation of persons with no hospital admission. Count data models were tested, and a clustered zero-inflated negative binomial model considered most valid statistically.

For the clustered zero-inflated negative binomial model, we used a logit model to assess odds ratios (ORs) for being at risk of any hospital admission. In the binomial part of the model, incidence rate ratio (IRR) was used to study the influence of increasing the explanatory factors by one unit for those at risk of any hospital admission. We used the same set of variables for both parts of the model, clustered on primary care practices, and adjusted for age and sex [18]. Predicted mean days hospitalised for the entire population were calculated as average marginal effects, combining the logit and the negative binomial parts of the model.

## Results

### Descriptive statistics

In the study population 13 122 persons were admitted to hospital during year 2007. For the population, total number of days hospitalised was 135 297 days and mean days hospitalised was 0.89. A total of 98 600 of the population were actively listed and 9.9% of actively listed where admitted to hospital compared to 6.3% of passively listed. Mean days hospitalised for actively listed were 1.05 and for passively listed 0.59 days. A total of 118 759 of the population consulted a GP once or less in 2007 and 884 of the population more than 7 times, with in mean 0.67 and 4.85 days hospitalised respectively.

A total of 7129 persons were diagnosed with at least one psychiatric disorder. No psychiatric disorder was found within RUB 0–1. RUB 3 was the most common multimorbidity level for all categories of psychiatric disorders. Of persons with any psychiatric disorder, 23.3% were admitted to hospital. Mean days hospitalised for persons with any psychiatric disorder were 4.95 compared to 0.69 for persons without any psychiatric disorder (Table 1).

**Table 1.** Descriptive for the population of Blekinge in 2007 (*N* = 151 731).

Descriptive	Group size		Admitted to hospital		Hospitalisation for the group		Hospital days
	*n*	%	*n*	%	Total days	%	Mean
No psychiatric disorder	144 602	95.3	11 463	87.4	100 139	74.0	0.69
Any psychiatric disorder	7 129	4.7	1 659	12.6	35 323	26.0	4.95
Psychoses	193	0.1	55	0.4	2 466	1.8	12.77
Depressive disorders	2 348	1.6	549	4.2	12 513	9.2	5.33
Anxiety disorders	2 133	1.4	396	3.0	6 685	4.9	3.13
Others	2 455	1.6	659	5.0	13 659	10.1	5.56
Passively listed	53 131	35.0	3 338	25.4	31 484	23.3	0.59
Actively listed	98 600	65.0	9 784	74.6	103 978	76.7	1.05
0 or 1 consultation	118 759	78.3	8 269	63.0	79 979	59.1	0.67
2 or 3 consultations	23 981	15.8	3 003	22.9	32 822	24.3	1.37
4 or 5 consultations	6 367	4.2	1 184	9.0	13 545	10.0	2.13
6 or 7 consultations	1 740	1.2	392	3.0	4 828	3.6	2.77
8- consultations	884	0.6	274	2.1	4 288	3.2	4.85
RUB 0	60 911	40.1	22	0.2	289	0.0	0.00
RUB 1	20 586	13.6	1 340	10.2	5 746	4.2	0.28
RUB 2	33 551	22.1	2 153	16.4	14 108	10.4	0.42
RUB 3	32 651	21.5	7 133	54.4	66 690	49.3	2.04
RUB 4	3 398	2.2	1 965	15.0	31 564	23.3	9.29
RUB 5	634	0.4	509	3.9	17 065	12.6	26.92
Women	75 087	49.5	7 078	53.9	73 455	54.3	0.98
Men	76 644	50.5	6 044	46.1	62 007	45.7	0.81
0–19 years	33 096	21.8	1 179	9.0	8 308	6.1	0.25
20–39 years	35 297	23.3	3 651	27.8	26 600	19.7	0.75
40–59 years	39 667	26.1	2 285	17.4	21 707	16.0	0.55
60–79 years	33 786	22.3	3 725	28.4	46 604	34.4	1.38
80+ years	9 885	6.5	2 282	17.4	32 243	23.8	3.26
Population of Blekinge	151 731		13 122		135 297		0.89

Unadjusted hospitalisation during 2007 distributed on explanatory factors for the population of Blekinge.

### Clustered zero-inflated negative binomial model

Odds of any hospital admission were 0.67 (95%CI 0.58–0.76) for persons actively listed compared to passively listed, 0.41 (95%CI 0.31–0.50) with 2–3 consultations in primary care compared to those with less. For all psychiatric disorders odds of any hospital admission were less than 1.00. For psychoses odds of any hospital admission was 0.77 (95%CI 0.47–1.08) and anxiety disorders 0.78 (95%CI 0.65–0.92) compared to persons without psychiatric disorder. At risk of any hospitalisation, mean days hospitalised was 0.76 (95%CI 0.72–0.79) without psychiatric disorder. All psychiatric disorders increased mean days hospitalised from anxiety disorders with 1.63 (95%CI 1.23–2.03) days to psychoses with 4.67 (95%CI 2.77–6.57) days (Table 2).

**Table 2.** Clustered zero-inflated negative binomial regression for the population of Blekinge in 2007 (*N* = 151 731). Mean days hospitalised calculated as average marginal effects.

Multivariate regression	Odds ratio for any hospital admission, population		Rate ratio of days hospitalised, population at risk		Mean days hospitalised, population	
	OR	(95%CI)	IRR	(95%CI)	Days	(95%CI)
No psychiatric disorder	1.00		1.00		0.76	(0.72–0.79)
Psychoses	0.77	(0.47–1.08)	7.05	(4.97–10.01)	4.67	(2.77–6.57)
Depressive disorders	0.81	(0.69–0.92)	2.81	(2.43–3.26)	1.90	(1.61–2.18)
Anxiety disorders	0.78	(0.65–0.92)	2.45	(1.97–3.05)	1.63	(1.23–2.03)
Others	0.90	(0.74–1.07)	2.37	(1.98–2.82)	1.69	(1.32–2.07)
Passively listed	1.00		1.00		1.23	(1.09–1.37)
Actively listed	0.67	(0.58–0.76)	0.84	(0.77–0.93)	0.86	(0.81–0.92)
0 or 1 consultation	1.00		1.00		1.16	(1.08–1.23)
2 or 3 consultations	0.41	(0.31–0.50)	0.89	(0.83–0.96)	0.74	(0.67–0.81)
4 or 5 consultations	0.53	(0.40–0.67)	0.76	(0.70–0.84)	0.68	(0.62–0.75)
6 or 7 consultations	0.51	(0.32–0.70)	0.74	(0.61–0.89)	0.65	(0.54–0.77)
8- consultations	0.73	(0.47–1.00)	0.82	(0.65–1.03)	0.82	(0.57–1.07)
RUB 0	1.00		1.00		0.00	(0.00–0.01)
RUB 1	6.63	(5.93–7.33)	0.29	(0.15–0.59)	0.31	(0.27–0.36)
RUB 2	6.71	(6.00–7.43)	0.39	(0.21–0.71)	0.44	(0.40–0.49)
RUB 3	8.28	(7.60–8.96)	0.51	(0.27–0.95)	2.00	(1.87–2.12)
RUB 4	9.97	(9.29–10.66)	0.93	(0.50–1.76)	8.14	(7.47–8.80)
RUB 5	11.26	(10.58–11.95)	1.79	(0.95–3.38)	20.13	(17.38–22.87)
Women	1.00		1.00		0.90	(0.84–0.96)
Men	1.14	(1.09–1.19)	0.99	(0.94–1.04)	0.96	(0.89–1.03)
0–19 years	1.00		1.00		0.55	(0.49–0.61)
20–39 years	1.96	(1.85–2.07)	0.98	(0.90–1.08)	0.95	(0.85–1.05)
40–59 years	1.01	(0.87–1.14)	1.04	(0.93–1.17)	0.58	(0.52–0.64)
60–79 years	1.10	(0.98–1.23)	1.60	(1.44–1.78)	0.94	(0.88–1.01)
80+ years	1.66	(1.53–1.78)	1.89	(1.69–2.12)	1.55	(1.42–1.68)

OR: odds ratio; IRR: incidence rate ratio; CI: confidence interval; RUB: Resource Utilization Band; *p* < 0.01 when significance; Odds ratio of hospitalisation: logit part of the regression; Rate ratio of days hospitalised: negative binomial part of the regression including only the population at risk; Mean days hospitalised: calculated average marginal effects for the population, combining both parts of the zero-inflated negative binomial model.

**Figure 1.**
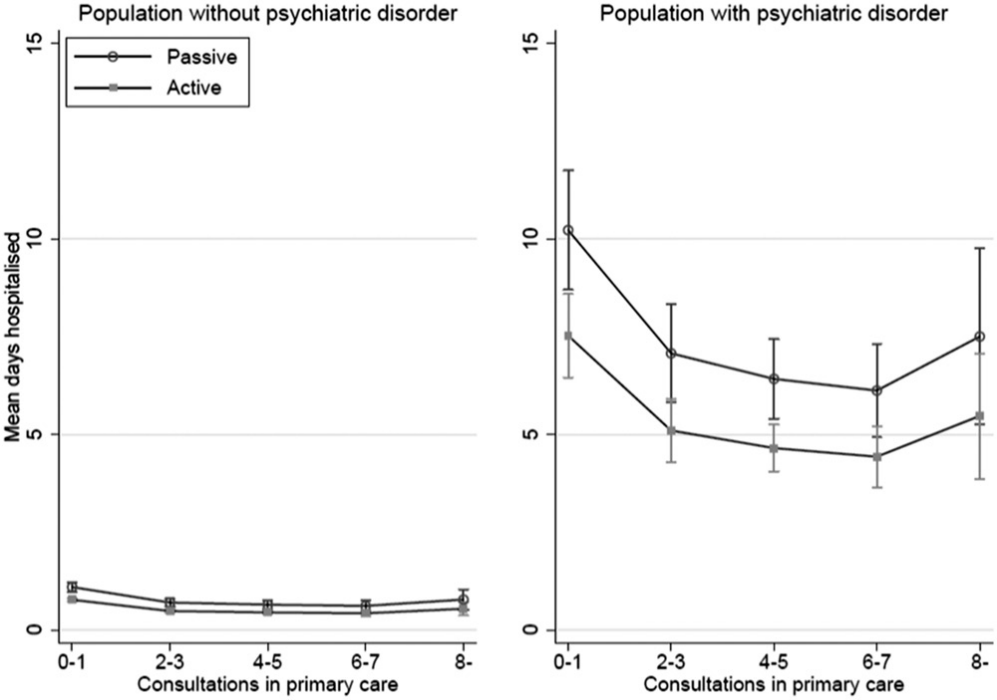
Predicted mean days hospitalised according to listing status and number of consultations in primary care for the population without (*N* = 144 602) and with (*N* = 7 129) psychiatric disorders, adjusting for multimorbidity level, age and sex.

Predicted mean days hospitalised for persons without psychiatric disorder with 0–1 consultations were 0.77 (95%CI 0.73–0.81) for actively listed and 1.10 (95%CI 0.98–1.21) for passively listed, and with 4–5 consultations 0.45 (95%CI 0.40–0.49) for actively and 0.65 (95%CI 0.54–0.75) for passively listed (Table 3) (Figure 1). Predicted mean days hospitalised for persons with any psychiatric disorder with 0–1 consultations were 7.53 (95%CI 6.46–8.60) for actively listed and 10.23 (95%CI 8.70–11.75) for passively listed, and with 4–5 consultations 4.65 (95%CI 4.05–5.26) for actively and 6.42 (95%CI 5.41–7.44) for passively listed (Table 3) (Figure 1).

**Table 3.** Predicted mean days hospitalised according to listing, number of consultations in primary care and multimorbidity level for the population without (*N* = 144 602) and with (*N* = 7 129) psychiatric disorders.

Adjusted mean days hospitalised	Actively listed		Passively listed	
	Days	(95%CI)	Days	(95%CI)
*Population without psychiatric disorder*				
0 or 1 consultation	0.77	(0.73–0.81)	1.10	(0.98–1.21)
2 or 3 consultations	0.48	(0.44–0.52)	0.70	(0.60–0.80)
4 or 5 consultations	0.45	(0.40–0.49)	0.65	(0.54–0.75)
6 or 7 consultations	0.42	(0.34–0.50)	0.61	(0.47–0.75)
8- consultations	0.54	(0.37–0.71)	0.78	(0.51–1.04)
*Population with psychiatric disorders*				
0 or 1 consultation	7.53	(6.46–8.60)	10.23	(8.70–11.75)
2 or 3 consultations	5.10	(4.30–5.90)	7.08	(5.82–8.33)
4 or 5 consultations	4.65	(4.05–5.25)	6.42	(5.41–7.44)
6 or 7 consultations	4.43	(3.65–5.21)	6.12	(4.92–7.32)
8- consultations	5.47	(3.87–7.08)	7.51	(5.25–9.76)
*Population without psychiatric disorder*				
RUB 0	0.00	(0.00–0.01)	0.01	(0.00–0.01)
RUB 1	0.25	(0.21–0.28)	0.39	(0.32–0.46)
RUB 2	0.35	(0.31–0.39)	0.56	(0.49–0.62)
RUB 3	1.64	(1.50–1.77)	2.41	(2.22–2.60)
RUB 4	6.97	(6.45–7.50)	9.09	(7.97–10.20)
RUB 5	17.53	(15.37–19.69)	21.53	(17.79–25.27)
*Population with psychiatric disorders*				
RUB 2	0.70	(0.56–0.84)	1.12	(0.89–1.34)
RUB 3	3.45	(2.84–4.07)	5.17	(4.36–5.98)
RUB 4	16.37	(14.04–18.69)	21.75	(18.58–24.93)
RUB 5	43.93	(35.77–52.10)	54.52	(43.64–65.41)

Adjusted mean days hospitalised calculated as marginal effects from the multivariate negative binomial model adjusted also for consultations, age and sex; Mean days hospitalised calculated separately according to listing status for the populations with and without psychiatric disorders; None with psychiatric disorder in RUB 0–1; CI: Confidence Interval.

**Figure 2.**
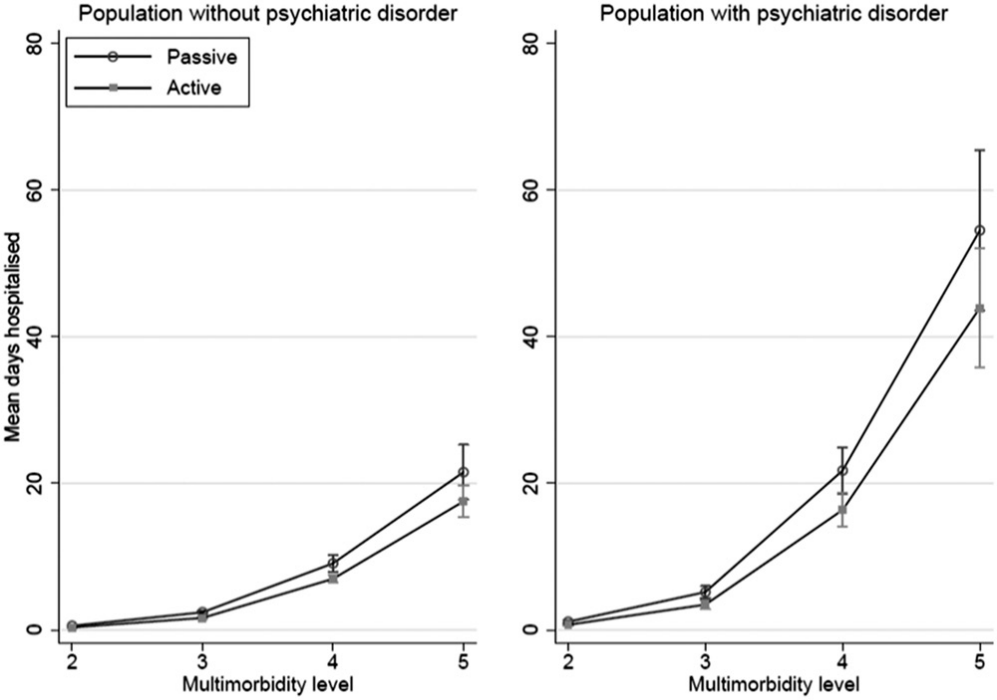
Predicted mean days hospitalised according to listing status and multimorbidity level for the population without (*N* = 144 602) and with (*N* = 7 129) psychiatric disorders, adjusting for number of consultations, age and sex.

At RUB 3, actively listed were in mean hospitalised for 3.45 (95%CI 2.84–4.07) days if diagnosed with any psychiatric disorder and 1.64 (95%CI 1.50–1.77) days if not. At RUB 3 passively listed were in mean hospitalised for 5.17 (95%CI 4.36–5.98) days, if diagnosed with psychiatric disorder and 2.41 (95%CI 2.22–2.60) if not (Table 3) (Figure 2).

## Discussion

Good patient-provider relationships, i.e. number of consultations and active listing, in primary care were associated with decreased mean days hospitalised, accounting for multimorbidity level, age and sex.

Active listing and more than mean consultations in primary care had a stronger association with decreased mean days hospitalised when psychiatric diagnoses contributed to the morbidity burden compared to less complex diagnostic patterns with the same need for health care.

### Strengths and weaknesses of the study

Data from all parts of a coherent Swedish healthcare system was used to study mean days hospitalised as an outcome of patient-provider relationships in primary care. The listing system allowed for identification of reasonable proxies of patient-professional relationships in patient records. A summary measure regarding all coexistent diagnoses and weighting different patterns of diagnoses was preferred as a measure of morbidity burden. We used ACGs to group different patterns of morbidity into six levels with the same need for health care within each level. This gave an opportunity to compare more complex patterns of diagnoses, i.e. including psychiatric diagnoses, with less complex patterns with the same need for health care.

Active listing could be regarded as patients acting to maintain their relationship with primary care. This interpretation of active listing depends on knowledge on settings and regulations of the listing system. To investigate this further requires qualitative research on the choice of primary care provider. The unadjusted association between higher mean days hospitalised for actively listed than passively listed is mediated by a higher multimorbidity level for actively listed compared to passively listed. Using listing as a measure of relationships underestimated good relationships, since that could be maintained by passive listing if patients’ do not protect their relationships with primary care. This diminished the associations between active listing and mean days hospitalised. Listing status was unlikely to be changed from active to passive according to listing regulations. Reliable data on listing was only available on practices preventing us from analyses of listing on individuals as GPs.

Consultations require both need for care, request and availability of care. Within this healthcare system availability of care was the same, and number of consultations in primary care could be regarded as a measure of aspects of the relationship between the population and primary care when accounting for morbidity burden. Number of consultations is related to trust in healthcare and other aspects of social capital [6], morbidity and the primary care system [8,19,20]. We considered more than mean consultations as having a relationship with primary care. Reliable data on consultations were only available for GPs which prevented analyses of other consultations in primary care.

The settings of this study were a small, comparatively simply organised healthcare, with a large enough population, and a listing system that is still valid. The County of Blekinge in 2007 provided us with a listing system comparable with contemporary Swedish primary care [12,13]. Competition and market economy principles were introduced in primary care in Blekinge in 2004 and legislated in Swedish primary care in 2010. Compared to most European primary care, Swedish primary care is known to be weak [19,21–23]. Today, Swedish primary care is characterised by adaptation to the legislation and an increased lack of continuity [24]. Generalisation of our results depend on analyses of similarities between healthcare and listing systems, that allows the interpretation of listing status and number of consultations as measures of aspects of relationships in primary care.

We analysed mean days hospitalised as an outcome of relationships with primary care. The cross-sectional design and statistical methods of this study allowed us to analyse associations, not causality. We could adjust for multimorbidity burden using all diagnoses from all health care but not for factors associated with secondary care and the decisions on admission and length of hospitalisation made within secondary care. Mean days hospitalised during a set period of time is more related to total need for hospital care for the population than number of hospital admissions. Thus mean days hospitalised is considered a better measure of the impact of primary care on other parts of the healthcare system than number of hospital admissions. Alternative explanations of our results could be associated with underlying factors related to patients as socioeconomic factors and social capital, organization of health care and factors related to secondary care. Our use of a coherent Swedish healthcare system tried to control for such factors. Mean days hospitalised and relationships with primary care have recently been studied for this population accounting for socioeconomic factors [25] showing decreasing mean days hospitalised with active listing and more consultations in primary care.

### Findings in relation to other studies

Whether primary care could reduce need for hospitalisation has been argued for a long time. Reid et al. [26] showed in 1999 that socioeconomic status was a major contributor to variation in hospitalisation between primary care practices while function of practices was not. In year 2003 a Swedish study analysed how the rates of GP consultations was related to hospitalisation accounting for socioeconomic factors and healthcare structure [27]. The conclusion was that a high rate of GP consultations was associated with less hospitalisation, as were socioeconomic factors and healthcare structure. Studies on predictors of high quality primary care have also stated that longer consultations and good teamwork are important for quality of care [28,29]. Previously we have studied mean days hospitalised as an outcome of primary care, accounting for socioeconomic factors also analysing difference between primary care practices [25]. Both our studies show that active listing and more than mean consultations in primary care lower mean days hospitalised, adjusting for morbidity burden. Our previous study showed a difference within primary care associated with ability to lower odds of any hospital admission [25]. This study showed that for both proxies of relationships with primary care the decrease in mean days hospitalised were stronger when psychiatric diagnoses added to the complexity of the morbidity burden.

A previous Swedish cohort study found elevated mortality in patients diagnosed with schizophrenia associated with undetected somatic diseases. Socioeconomic status and medical treatment of patients were considered, but not factors related to primary care [30,31]. In a previous study we showed that socioeconomic factors were less associated with mean days hospitalised than relationship with primary care [25]. This paper shows that multimorbidity including psychiatric disorders increased mean days hospitalised, and that relationships with primary care lower mean days hospitalised more for those with psychiatric diagnoses than those without. All groups of psychiatric disorders lowered odds of any admission to hospital, while mean days hospitalised increased both for those at risk of hospitalisation and for the population. The association with mean days hospitalised was stronger for psychoses than other psychiatric disorders. The inconsistency between odds of any hospitalisation and days hospitalised for psychiatric disorders might be associated both with propensity to trust, trustability [6] and undetected somatic diseases [29,30]. To study this further more data, a larger population and mixed methods would be needed.

Aggregated data from 34 countries (including Scandinavia) was used to investigate the association between characteristics of primary care and diabetes-related hospitalisation. The conclusion was that it takes more than strong primary care to avoid hospitalisation [32]. We did not study avoidable hospitalisation or availability of hospital beds. Instead, we analysed the association between relationships with primary care, patient complexity and all cause hospitalisation using almost the same statistical model. Our population was part of the aggregated data and both secondary and primary care were uniform in organisation and availability. With no difference in strength of primary care and adjusting for multimorbidity, our study found that good relationships in primary care are associated with less mean days hospitalised, stronger with more complex patterns of multimorbidity.

### Meaning of the study and conclusions

We conclude that good patient-provider relationships, i.e. more than mean consultations and active listing, with primary care are associated with decreasing mean days hospitalised accounting for morbidity burden, age and sex. The impact of patient-provider relationships in primary care on mean days hospitalised increased when psychiatric disorders added to patient complexity.

Promoting good relationships with primary care might be an option to decrease need for hospitalisation, especially for patients with more complex diagnostic patterns.

We analyse mean days hospitalised as an outcome of patient-provider relationships with primary care. To include the decision to hospitalise, factors related to individual hospital admissions and other factors related to secondary care will add to the analyses of healthcare systems. This requires mixed methods and is not within the aim of this paper. How length of hospitalisation is related to the interaction between socioeconomic status, relationships in primary care and patient complexity need to be studied further. To further understand need for hospitalisation for patients with psychiatric disorders, care for psychiatric and somatic disorders need to be studied separately, including relationships with primary care.
